# Warming mediates the relationship between plant nutritional properties and herbivore functional responses

**DOI:** 10.1002/ece3.2602

**Published:** 2016-11-17

**Authors:** Meng Xu, Jaimie T. A. Dick, Anthony Ricciardi, Miao Fang, Canyu Zhang, Dangen Gu, Xidong Mu, Du Luo, Hui Wei, Yinchang Hu

**Affiliations:** ^1^ Pearl River Fisheries Research InstituteChinese Academy of Fishery SciencesKey Laboratory of Tropical and Subtropical Fishery Resource Application and CultivationMinistry of AgricultureGuangzhouChina; ^2^ Institute for Global Food SecuritySchool of Biological SciencesQueen's University Belfast, MBCBelfastUK; ^3^ Redpath MuseumMcGill UniversityMontrealQCCanada; ^4^ College of Fisheries and Life ScienceShanghai Ocean UniversityShanghaiChina

**Keywords:** ecological impacts, ecological stoichiometry, functional responses, increasing temperature, invasive herbivore species, metabolic theory of ecology, nutritional property, per capita effect

## Abstract

Quantifying the per capita effects of invasive alien species is crucial for assessing their ecological impact. A major challenge to risk assessment of invasive species was to understand the factors that cause per capita effects to vary in different ecological contexts, particularly in a warming world. By conducting functional response experiments, we estimated the per capita effects (attack rate and maximum feeding rate) of an invasive herbivorous snail, *Pomacea canaliculata*, toward ten host plant species. We tested whether variation in these effects is related to plant nutritional and physical properties (total N and dry matter content (DMC)) and examined how increasing temperature can shift these relationships. We observed stronger per capita effects (i.e., higher attack rate and maximum feeding rate) by the snail on plants with higher total N, but no direct relationship was found with DMC. A significant interaction effect of total N and DMC on the attack rate indicated that DMC probably adjusted the feeding indirectly. Warmer temperatures reduced correlations between snail functional responses and host plant nutritional properties (total N) by increasing maximum feeding rate for plants of low nutrition, but there was no such effect on attack rates. However, given the nonreplacement design used in our study, the nonsignificant effect of temperature on the attack rate should be caveated. Our result suggests that characterizing the per capita effects of herbivores using functional responses can reveal the mechanisms by which climate change may alter herbivore–plant interactions and, thus, the ecological impacts of introduced herbivores.

## 1 Introduction

A predictive understanding of the ecological impacts of invasive alien species is a central goal of invasion ecology (Pyšek & Richardson, 2010; Ricciardi, Hoopes, Marchetti, & Lockwood, 2013). In order to facilitate the comparison of information between regions and to allow for the objective prioritization of species management actions, better metrics for quantifying impact must be developed and applied (Pyšek & Richardson, 2010). Functional response (FR) analysis is a promising new method to understand the impact of invaders, providing important insights into consumer–resource interactions and per capita effects (Dick et al., 2014), and has been used to explain and predict the ecological impacts of alien predators (Alexander, Dick, Weyl, Robinson, & Richardson, 2014; Bollache, Dick, Farnsworth, & Montgomery, 2008; Dick et al., 2013; Dodd et al., 2014). Recently, this method has been extended to predict the potential impacts of alien herbivores (Xu et al., 2016), but it is still underapplied to herbivores, in spite of offering a convenient experimental design and meaningful mechanistic understanding of resource consumption.

The invasive golden apple snail (*Pomacea canaliculata*; Gastropoda, Ampullariidae) is a model species for investigating the success and impact of invaders (Hayes et al., 2015). Its invasions have resulted in acute grazing damage to agriculture and wetland macrophytes (Burlakova, Karatayev, Padilla, Cartwright, & Hollas, 2009; Carlsson & Lacoursiere, 2005; Cowie, 2002; Wong, Liang, Liu, & Qiu, 2010) and affected water quality and ecosystem functioning (Carlsson, Brönmark, & Hansson, 2004). Serious damage to agriculture and the economy has led to its listing in “100 of the World's Worst Invasive Alien Species” (Lowe, Browne, Boudjelas, & De Poorter, 2000). Given its substantial grazing impact, several studies have analyzed the consumption of macrophytes by *P. canaliculata* (Boland et al., 2008; Estebenet, 1995; Lach, Britton, Rundell, & Cowie, 2000), feedbacks on the snail itself (Estoy et al., 2002; Qiu & Kwong, 2009; Tamburi & Martín, 2011), and the causes that drive its food preferences (Burlakova et al., 2009; Qiu & Kwong, 2009; Wong et al., 2010). However, the majority of these studies used “snapshot” assessments of resource uptake per unit time, rather than characterizing the relationship between resource availability and resource consumption—that is, its functional response (FR) (Holling, 1959). The problems with choosing one particular level of resource availability, or providing the resource in excess, is that the per capita effect of consumer impact (such as attack rate, handling time, and maximum feeding rate) cannot be characterized and that differences in consumer impact may be missed as no opportunity is given for FR types and magnitudes to emerge and perhaps diverge (Dick et al., 2014).

Ecological stoichiometry theory (EST) describes how the chemical elements and their ratios determine individual consumer–resource interactions, population dynamics, and community and ecosystem patterns (Sterner & Elser, 2002). In previous studies, the feeding preferences and efficiencies of *P. canaliculata* were already linked to stoichiometry and physical properties of plant resources (Qiu & Kwong, 2009; Wong et al., 2010). For example, Wong et al. (2010) found that the feeding rate of *P. canaliculata* was positively related to total nitrogen content and negatively related to the ratio of carbon to nitrogen and dry matter content (DMC). However, given that snapshot measurements of feeding do not necessarily predict the feeding ability of a herbivore, it remains unknown whether plant properties influence critical elements of feeding efficiency—that is, attack rates, handling times, and estimated maximum feeding rates. Meanwhile, temperature affects the feeding as also predicted by the metabolic theory of ecology (MTE, Brown, Gillooly, Allen, Savage, & West, 2004), thereby potentially altering consumer–resource interactions, population dynamics, and food web structure (Vucic‐Pestic, Ehnes, Rall, & Brose, 2011; Ott, Rall, & Brose, 2012; Rall et al., 2012; Sentis, Hemptinne, & Brodeur, 2013; but see Englund, Ohlund, Hein, & Diehl, 2011; Lemoine & Burkepile, 2012). Further, increasing temperature can increase the rate of protein denaturing, protein turnover rate, and significant respiratory and nitrogen utilization costs (Lemoine & Shantz, 2016); rising temperature probably increases consumer growth rates and thus potentially enhances the demand for phosphorus‐rich materials as well (Lemoine, Drews, Burkepile, & Parker, 2013 and references therein). While temperature may interact with resource stoichiometry to influence consumer–resource interactions and potential population dynamics (Allen & Gillooly, 2009; Ott et al., 2014), to date it has been rare for studies to attempt to examine how the FR depends on the combined effects of warming and the resource properties (but see Ott et al., 2012).

In this study, we firstly analyzed the functional responses of the invasive golden apple snail *P. canaliculata* toward 10 field and cultivated plants that are common resources of the snail (Qiu & Kwong, 2009). Then, we relate per capita effects to physicochemical characteristics (total N and DMC) of these plants to analyze the potential causes associated with differential per capita impacts. Finally, we examined how temperature may alter the influence these plant properties on per capita effects and hence ecological impact.

## 2 Materials and Methods

The study was conducted at the Pear River Research Institute (23°04′9.42″N; 113°12′52.68″E) in Guangzhou City of China in 2015. The golden apple snail, *P. canaliculata*, was cultured in ponds (5 m × 4 m × 1.5 m) with recirculating water systems and fed with carp food pellets (composing of crude protein, amino acid, fat, calcium, and phosphorus; Zhongshan President Enterprises Co., Ltd, Zhongshan, China). We chose ten plant species as food resources, namely *Apium graveolens* (Chinese celery), *Alternanthera philoxeroides* (alligator weed), *Colocasia esculenta* (taro), *Eichhornia crassipes* (water hyacinth), *Hydrocotyle vulgaris* (pennywort), *Ipomoea batatas* (sweet potato), *Ipomoea aquatica* (water spinach), *Lactuca sativa* (lettuce), *Myriophyllum aquaticum* (parrot feather), and *Pistia stratiotes* (water lettuce) (Table 1). We collected fresh leaves of these species around the Peal River Fisheries Research Institute on the day the experiment started in order to make the leaves as fresh as possible. These plants are common resources for *P. canaliculata*, and they are commonly used in the feeding trials before (Qiu & Kwong, 2009; Wong et al., 2010). When using the fresh leaves of the same plant species as these papers, we assumed that the properties of these plants are also the same as those and denoted the important identity of plants per se. Although the potential wilting or age difference may influence the DMC slightly, the relatively large interspecific variations among these species probably make these potential intraspecific differences less sensible. In this study, thus, we did not measure the nutritional and physical properties of these plants again; rather, we collected them from both literatures of one research team. Most of the properties were collected directly from Wong et al., 2010; while those for the *A. graveilens* were from Qiu & Kwong, 2009. For the *Ipomoea*, while we only obtained the properties of *I. aquatic*, we assumed that two congeneric species *I. aquatic* and *I. batatas* had same properties.

**Table 1:** Nutritional and physical properties of 10 plants used in the experiment (mean ± standard deviation)

Plant Species	Habit	DMC (%)	N (%)
*Alternanthera philoxeroides*	Aquatic	21.7 ± 0.3	5.2 ± 0.7
*Eichhornia crassipes*	Aquatic	15.0 ± 0.6	2.9 ± 0.1
*Hydrocotyle vulgaris*	Aquatic	17.6 ± 2.5	5.3 ± 0.1
*Myriophyllum aquaticum*	Aquatic	19.7 ± 1.6	3.6 ± 0.0
*Pistia stratiotes*	Aquatic	6.3 ± 0.4	2.5 ± 0.1
*Apium graveolens*	Semi‐aquatic		4.8 ± 0.1
*Colocasia esculenta*	Semi‐aquatic	21.6 ± 0.3	2.9 ± 0.1
*Ipomoea aquatic*	Aquatic	10.2 ± 0.6	6.5 ± 0.0
*Ipomoea batatas*	Semi‐aquatic	10.2 ± 0.6	6.5 ± 0.0
*Lactuca sativa*	Semi‐aquatic		4.7 ± 0.7

The data of DMC (dry matter content) and total N are from published literatures (Qiu & Kwong, 2009; Wong et al., 2010).

### 2.1 Functional response experiment

We conducted a functional response feeding experiment in a recirculating water system that includes five tanks (each tank: 200 cm × 70 cm × 50 cm) in the summer of 2015 and ran this experiment in three consecutive blocks. The first block started at 08:00 on May 29 and ended at 20:00 on May 31 (60 hr), and similarly, the second and third blocks started at 08:00 on June 4 and June 10, respectively, and ended at 20:00 on June 6 and June 12, respectively. Before the experiment, the snails were held without food for 24 hr to allow for standardization of hunger levels, a common method in functional response experiments (Alexander et al., 2014; Xu et al., 2016). Snails with similar body size (mean body mass ± *SE*: 13.04 ± 0.05 g) were used to minimize variation resulting from different body masses (Rall et al., 2012). Fresh leaves of all 10 plants were weighed and allocated to experimental units. The temperature over the experimental duration was 22–26°C at nighttime and 26–32°C during daytime.

For each block, a split‐plot design was used. We had five whole plot units (the tank) with water temperature controlled at 26, 28, 30, 32, and 34°C. We heated the tanks using water tank heaters and cooled the water by diluting the tank water using tap water. Within each tank, 70 plastic boxes (12 cm × 10 cm × 6 cm) were used as subplot experimental units. These 70 boxes consisted of the 10 plant species with each having seven biomass gradients (wet weight 1, 2, 4, 6, 8, 10, and 12 g) and were totally randomized in the tank. In each box, a golden apple snail was introduced. Collectively, the experiment included a total of 1050 experimental units (3 blocks × 5 temperatures × 10 plant species × 7 resource biomass). We measured plant consumption by snails as the difference between initial and final leaf wet weight.

### 2.2 Functional response analyses

Functional response (FR) analyses methods for this invasive herbivorous species have been developed and used in our previous studies (Xu et al., 2016). In this study, we used hyperbolic type II FRs and Rogers’ random predator equation to characterize the relationship between initial plant biomass and consumption by snails, which can better correct for the effects of resource depletion compared to the Holling's disk equation (Haddaway et al., 2012; Juliano, 2001; Rogers, 1972):

(1) $$N_{e}=N\left(1-\text{exp}\left(a\left(N_{e}h-T\right)\right)\right)$$

where *N*_*e*_ is the amount of resource consumed, *N* is the initial biomass, *a* is the “attack rate,” *h* is the “handling time,” and *T* is the total experiment time. This recursive function can be resolved using the *lambert W* function (Corless, Gonnet, Hare, Jeffrey, & Knuth, 1996):

(2) $$N_{e}=N-\frac{W(ahN\text{exp}(-a(T-hN)))}{ah}$$

We derived the FRs for the snail toward each of the 10 plant species at each experimental temperature. The parameters *a* and *h* for the FRs were estimated using nonlinear least‐squares regression and the *lambert W* function of the package “emdbook” (Bolker, 2010). The estimated maximum feeding rate was calculated as 1/*h*. The parameters of *a* and *h* in predator–prey systems are classically interpreted as the “attack rate” and “handling time,” respectively, but can also include other elements of consumption, such as digestion rate (Jeschke, Kopp, & Tollrian, 2002). In the context of herbivores, attack rates may translate as ingestion rates (the product of bite frequency and bite size) and handling times as chewing time (Farnsworth & Illius, 1996; Spalinger & Hobbs, 1992).

### 2.3 Statistical analyses

Using a restricted maximum‐likelihood (REML) method in the “lme” function of the “nlme” package (Pinheiro & Bates, 2006), we fitted linear mixed models (LMMs) to the functional response parameters (attack rate and maximum feeding rate). As both variables did not fit the normal distributions (shapiro.test: *P *<* *.0001 for both parameters), we used log transformation to make them fit better before including the LMMs (after transformation: *P *=* *.937, *P *=* *.053 for both parameters). In these models, properties of plant species (total N and DMC) and temperature were used as fixed effects. We tested their effects on the attack rate and maximum feeding rate, respectively. The tank was used as random effect, describing the error structure of a split‐plot design (Crawley^2012^). The notation of the model was as follows:

$$\begin{array}{cc} y= & \beta_{0}+\beta_{1}\text{temperature}+\beta_{2}N+\beta_{3}\text{DMC}+\beta_{4}\text{temperature:N}+\\ & \beta_{5}\text{temperature:DMC}+\beta_{6}N:\text{DMC}+\beta_{7}\text{temperature:N:DMC}+\\ & \mu_{\mathrm{tank}}+\varepsilon \end{array}$$

where *y* is the parameter derived from the type II functional response model (attack rate and maximum feeding rate). β_0_ is the intercept. β_1_, β_2_, and β_3_ are coefficients associated with temperature and properties of the plants. β_4_, β_5_, β_6_, and β_7_ are the coefficients characterizing the interactions among temperature, total N, and DMC. μ_tank_ is the coefficient of the random effect (tank) depicting the error structure of the split‐plot design and ε is the remaining variation. All statistical analyses were performed in R, version 3.3.1 (R Core Team 2016).

## 3 Results

**Figure 1:**
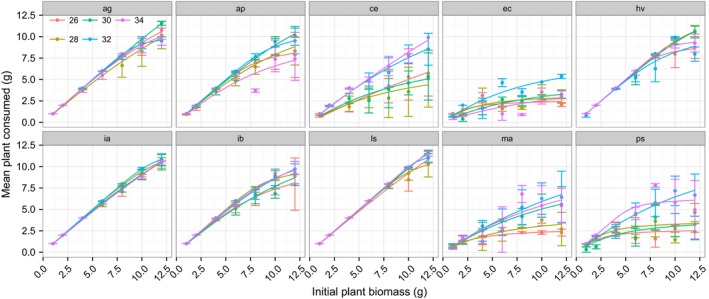
Type II functional responses of the invasive herbivorous snail *Pomacea canaliculata* toward 10 plant species at experimental temperatures of 26, 28, 30, 32, and 34°C. The abbreviations ag, ap, ce, ec, hv, ia, ib, ls, ma, and ps represent *Apium graveolens, Alternanthera philoxeroides, Colocasia esculenta*,* Eichhornia crassipes, Hydrocotyle vulgaris, Ipomoea aquatic, Ipomoea batatas*,* Lactuca sativa, Myriophyllum aquaticum,* and *Pistia stratiotes*, respectively. Error bars represent standard errors

**Figure 2:**
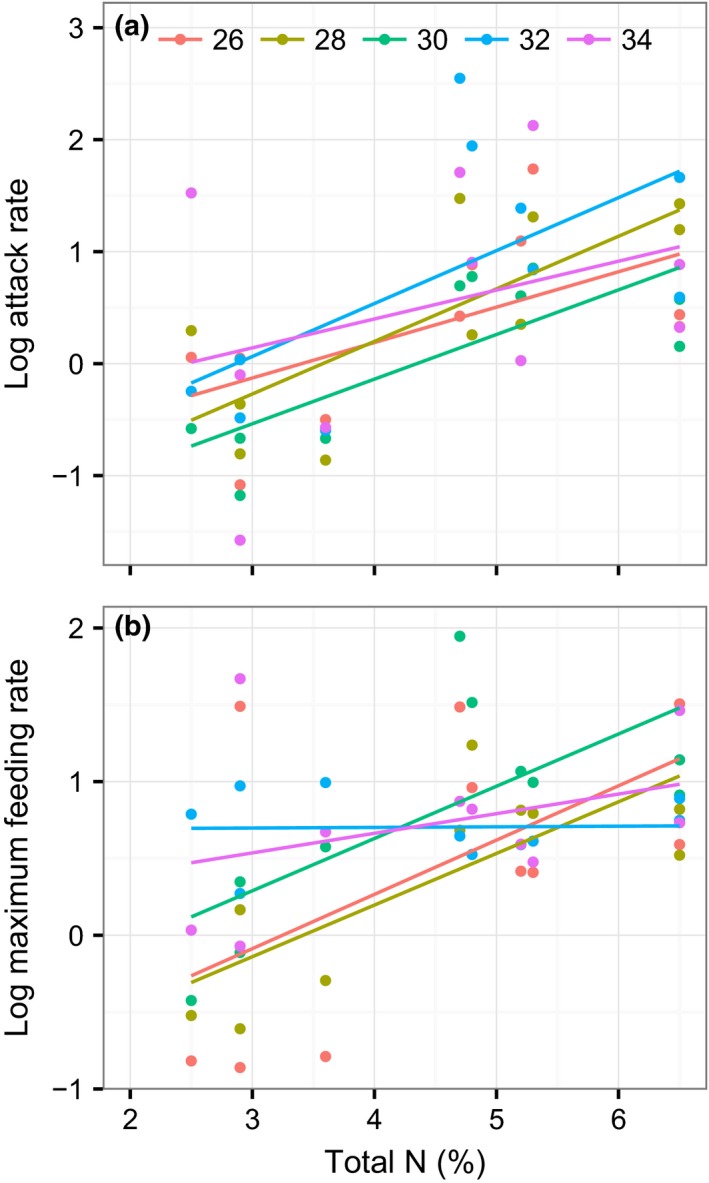
Relationships between total nitrogen of plants and attack rate (m^2^/day, a) and maximum feeding rate (g/day, b) of the herbivore at 26, 28, 30, 32, and 34°C. The linear fits come from two‐variable mixed models

**Figure 3:**
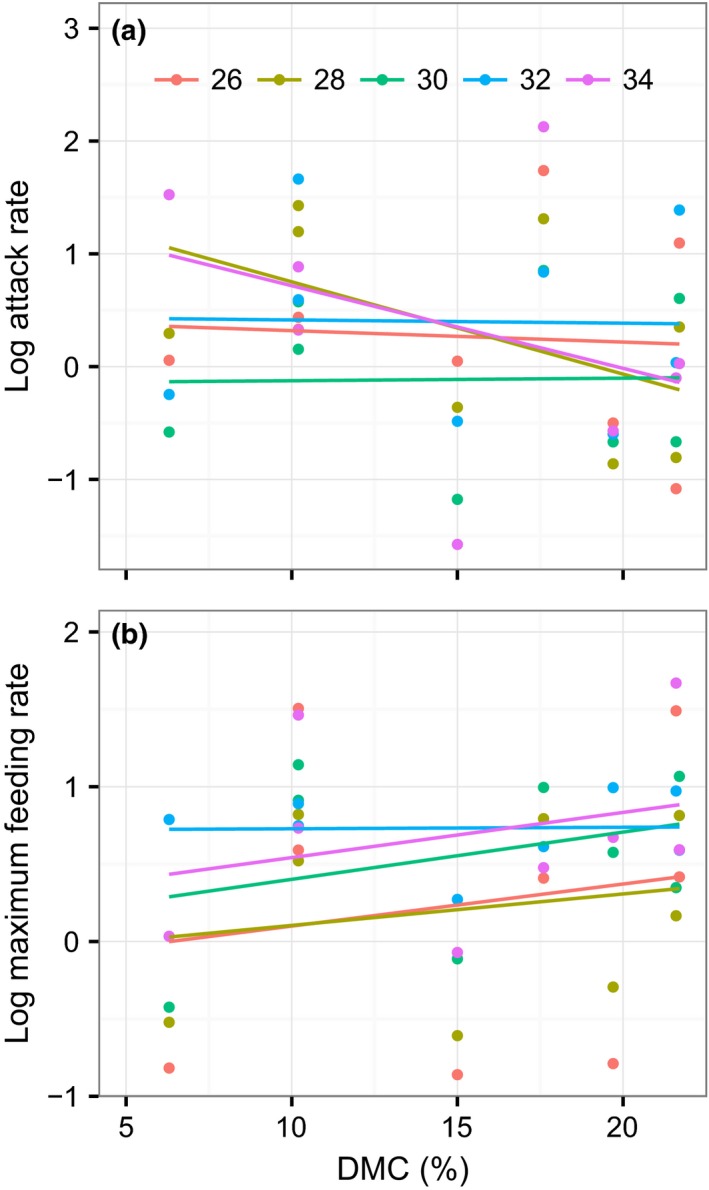
Relationships between DMC of plants and attack rate (m^2^/day, a) and maximum feeding rate (g/day, b) of the herbivore at 26, 28, 30, 32, and 34°C. The fitted lines were generated by two‐variable mixed models

There was substantial variation in snail FR across the 10 host plant species. *Apium graveolens, Hydrocotyle vulgaris, Ipomoea aquatic, Ipomoea batatas*, and *Lactuca sativa* were subject to markedly higher FRs from the snail (Figure 1). Overall, across all plant species, warming did not significantly influence FR parameters (Table 2). This result reflects the weak effects of temperature on species that were subject to high FRs and its strong enhancement of the snail FRs for other plants (Figure 1). Properties of the plants (total N, but not DMC) significantly predicted the FR of this invasive herbivore, with the snails preferring to feed on plants with high N (Table 2; Figures 2 and 3). There was a significant interaction effect of property of the plants (total N, but not DMC) and temperature for the maximum feeding rate as well (Table 2), with increasing temperature lowering the positive correlation of the maximum feeding rate with total N (Figure 2b). This pattern was mainly due to the increasing maximum feeding rate for plants of low nutrition with increasing temperature (Figure 2b). There were no interaction effects of plant properties and temperature for the attack rate (Table 2; Figures 2a and 3a), indicating that, in this study, the temperature did not affect nutritive dependence of the specific attack ability. However, given the nonreplacement design used in this study, the nonsignificant effect of temperature should be caveated. A significant interaction effect of total N and DMC on the attack rate was found, indicating an indirect role of DMC in adjusting the feeding. We also tested the significances of random effects for all the models and found no significant variances among different tanks (likelihood ratio test, all *P *>* *.05).

**Table 2:** Results of linear mixed models predicting the log response of attack rate (*a,* m^2^/day) and maximum feeding rate (max*,* g/day) derived from the type II functional responses to temperature and plant properties (total N and DMC of plants) .Significant results (*P* < .05) are shown in boldface type

Models and variables	*df*	Attack rate (*a*)		Maximum feeding rate (max)	
		*F*	*P*	*F*	*P*
DMC	29	5.118	.088	2.330	.138
N	29	27.588	**<.001**	21.409	**<.001**
Temperature	3	0.082	.794	6.817	.080
DMC:N	29	11.879	**.002**	2.957	.096
DMC:temperature	29	0.127	.724	0.023	.879
N:temperature	29	0.186	.669	4.312	**.047**
DMC:N:temperature	29	0.078	.783	0.075	.786

## 4 Discussion

Conventionally, the feeding ability of herbivores is measured either as plant consumption (Carlsson & Lacoursiere, 2005; Jogesh, Carpenter, & Cappuccino, 2008; Lemoine et al., 2013; Morrison & Hay, 2011; Parker & Hay, 2005; Wong et al., 2010) or inferred from a ratio of plant biomass with and without the herbivore present after some time (Deraison, Badenhausser, Börger, & Gross, 2015; Novak & Wootton, 2010; O'Connor, 2009). These “snapshot” measurements (measuring plant biomass at the end of the experiment) ignore the subtleties of the shape and magnitude of consumer–resource interactions, which are better characterized by the herbivore functional response; otherwise, we may draw incomplete or even incorrect conclusions when comparing the consumption rate among different species or among different situations for the same species (Dick et al., 2014). More importantly, the “snapshot” measurement gives limited mechanistic information on per capita feeding efficiency as broken down into attack rates, handling times, and estimated maximum feeding rates. Additionally, quantifying such per capita effects is a necessary yet understudied component of assessing the ecological impacts of alien invasive species (Dick et al., 2014; Parker, Simberloff, & Lonsdale, 1999; Ricciardi et al., 2013). It is surprising that this method is thus far scarcely used to measure the feeding efficiency and ecological impacts of herbivorous species in practice, although it has been verified theoretically (Farnsworth & Illius, 1996; Spalinger & Hobbs, 1992) and by us empirically (Xu et al., 2016).

Here, we found that the nutritional characteristics of plants can predict the per capita feeding rates of the invasive species *P. canaliculata*. Attack rate and maximum feeding rate are all significantly correlated with the total N content of plant tissues. These results are consistent with studies showing that herbivores favor plants possessing a high nutrient content (Lodge, 1991; Mattson, 1980) and that the nutritional quality of macrophytes influences their palatability to *Pomacea* and other native snail species (Elger & Lemoine, 2005; Qiu & Kwong, 2009; Wong et al., 2010). However, these “snapshot” studies rarely examined the FR and associated per capita feeding efficiency. Our experiments mechanistically showed that the high nutrient contents of plants result in them suffering higher attack rates and maximum feeding rates (low handling times), and thus render them vulnerable to more consumptive pressure from the golden apple snail.

Unlike other studies showing a negative correlation between plant DMC and palatability to native (Elger & Willby, 2003) and invasive *Pomacea* snails (Burlakova et al., 2009; Wong et al., 2010), and previous suggestions that DMC may be an important indicator of antiherbivore defenses (Burlakova et al., 2009; Elger & Willby, 2003), we did not find any significant relationships between DMC with attack rate and maximum feeding rate of this invasive species. While the DMC of plants may indeed affect total consumption by the herbivore after some period of time, it does not predict attack ability and feeding potential that may subtly change with other factors such as chemical defense of plants and physiological response of herbivores. Nevertheless, a significant interaction effect of DMC and total N on the attack rate indicated that DMC probably affected the feeding of the invasive herbivore indirectly. Our result emphasizes the importance of quantifying the FR, given that a discrete amount of consumption over a single time interval does not necessarily predict the potential maximum feeding rate (Dick et al., 2014).

Temperature can strongly influence consumer FR in such a way so as to alter the strength of consumer–resource interactions, population dynamics, and food web stability (Englund et al., 2011; Rall, Vucic‐Pestic, Ehnes, Emmerson, & Brose, 2010; Sentis, Hemptinne, & Brodeur, 2012; Vucic‐Pestic et al., 2011). The effect of temperature on FR parameters is commonly considered under the framework of MTE with respect to the body size of consumers (Ott et al., 2012; Rall et al., 2012; Sentis et al., 2013). Some studies, however, indicated that the MTE probably is not the general rule in predicting the FR parameters and consumption‐related rates (Englund et al., 2011; Lemoine & Burkepile, 2012). For our study, across all plant species, temperature did not significantly affect the maximum feeding rate in general, reflecting highly differential effects of temperature on the feeding behavior of this invasive herbivore toward different plants. Increased temperature significantly enhanced snail feeding efficiency only on plants that were otherwise subject to the lowest FRs owing to their nutritional properties; this result is indicated by a significant interaction between these properties and temperature. Increasing temperature reduced the correlations of total N with maximum feeding rate, as it increased maximum feeding rate for plants with low nutrition. While supporting the assertion that MTE did not completely predict the rate of consumer–resource interactions, our results further indicate that integrating the EST into MTE may shed more light on understanding species interactions, population dynamics, and food web stability (Allen & Gillooly, 2009; Ott et al., 2014).

Resource quality is rarely linked to temperature in explaining the changes of FR parameters (but see Ott et al., 2012), although these parameters reflect both consumer and resource characteristics rather than only the physiological properties of consumers (Jeschke et al., 2002; McCoy & Bolker, 2008). To compensate for increased nutritional demands at high temperature, herbivores may either feed preferentially on plants with high nutritional quality or, if adopting a compensatory feeding strategy, increase consumption of low‐quality plants (Lemoine et al., 2013; Ott et al., 2012). In our study, increasing temperature induced compensatory feeding, that is, increasing the per capita effects of this herbivorous snail on the plants with low nutrition, and thus eroded the correlation between plant nutrition and FR. Although several studies have demonstrated the relationship between plant palatability with plant nutritional conditions (Qiu & Kwong, 2009; Wong et al., 2010), fixed temperatures in such studies did not allow tests of the possible effects of changing environmental variables. Our results suggest that ignoring this important aspect may fundamentally undermine our ability to assess and predict the impacts of invasive herbivores. However, recent studies indicate that the increasing temperature also likely enhanced the effects of nutrient supply on growth rate (Cross, Hood, Benstead, Huryn, & Nelson, 2015) and thus does not necessarily result in strong nutrient limitation. To illuminate these potentially different predictions, it is required to compare and distinguish the metabolic rate, consumption rate, and growth rate scaling with increasing temperature (Lemoine & Burkepile, 2012), and to combine the MTE and EST to understand nutrient uptake under the context of climate change (Allen & Gillooly, 2009; Ott et al., 2014).

We found that increasing temperature mediated the effect of plant nutrition on maximum feeding rate, but not on attack rate. Attack rate and maximum feeding rate often represent fundamentally different processes, with maximum feeding rate being closely related to the physiology (e.g., enzyme activity) of the consumer, whereas attack rate largely reflects behavioral traits such as search and attack (Englund et al., 2011). In the context of herbivore FR, a bite of herbivore corresponds to the prey item of the classical predator–prey FR, and the attack rate corresponds to the product of bite frequency and bite size (g/bite) (Farnsworth & Illius, 1996; Spalinger & Hobbs, 1992). Thus, while the bite size is regulated by the morphology of the animal's mouth and the volume of the plant and its bulk density (Spalinger & Hobbs, 1992), the attack rate of herbivore FR may be regulated mainly by behavioral and morphological traits rather than physiology. Increasing temperature may mainly affect the physiological activity rather than the behavior and morphology of the snail, thus affecting the maximum feeding rate rather than the attack rate. However, as we used the nonreplacement design (without renewal of the depleted resource), where all biomass was consumed at the low levels of plant biomass for some plant species, the attack rate (the initial slope of FR curve) may be constrained and thus, potential effects of temperature were probably underestimated. Actually, a major issue with respect to deriving FR is the experimental design where resources are replaced or not replaced as they are consumed, although as used here, the Rogers’ random predator equation can help estimate parameters if not solve this problem completely (Alexander, Dick, O'Connor, Haddaway, & Farnsworth, 2012; Dick et al., 2014). However, due to the practical difficulties of replacing prey (or biomass) after consumption, the nonreplacement design and associated analysis with the random equation are still very common in the studies of predator FRs (but see Alexander et al., 2012). The replacement design is even more difficult for herbivore FRs due to the difficulty in obtaining the consumed biomass and replacing biomass during the course of the experiment. Nevertheless, the replacement should be advocated in FR experiments, and the nonsignificant result for the attack rate in this study should be caveated. Additionally, another critical aspects in FR curve fitting is the sufficient replication of satiation biomass (=density). If the experimental data do not include the biomass where satiation is reached, the estimates of handling time and associated maximum feeding rate might be affected. In our study, the high consumption of *P. canaliculata* on the *A. graveolens, I. aquatic, and L. sativa* rendered not enough satiation replications and thus likely influenced the estimates of maximum feeding rate for these plant species, but the general pattern remained in terms of high and low FR curves.

FR analysis of herbivorous consumers has been underapplied in ecology in general and for understanding invasive species impacts in particular (Dick et al., 2014). Our previous study, using alien invasive, alien noninvasive, and native snails, indicated that comparing the FR can predict the invasiveness and ecological impacts of alien herbivorous species (Xu et al., 2016), which is testified for alien predators as well (Alexander et al., 2014; Dick et al., 2013; Dodd et al., 2014). As species invasions may fundamentally alter interactions of food webs and biodiversity, the development of such a simple experimental method to analyze the strength and variance of herbivore–plant interactions is of potentially immense value to ecologists. Particularly, under the context of global change, as the interactions between invasive species and native resource may be altered or intensified, understanding the changes of FR under different stressors such as increasing temperature and element cycling may shed more light on the ecological impact assessment of alien species.

## Conflict of Interest

None declared.
